# Supplementary material for: Coalescence of multiple droplets induced by a constant DC electric field
Source: PLoS One. 2024 Apr 9;19(4):e0300925. doi: 10.1371/journal.pone.0300925 (PMC11003697; doi:10.1371/journal.pone.0300925)
Supplement: Attachment — Submitted filename: Response to Reviewer #2.doc [file pone.0300925.s002.doc]

**Response to Reviewer #2**

The Authors present an interesting investigation on the capabilities hold by DC electric fields in producing and sustaining electro-coalescence. The topic is very actual and interesting and most of the data the Authors report are sound. However, I have several concerns regarding the completeness of the work, the way in which it has been conducted and written, that force me to suggest to propose major revisions before re-considering the paper for eventual acceptance. Please find in the following the most important issues.

**Ans:** Thank you very much for your positive comments. We responded to your specific comments point by point below.

1. Abstract can be improved to a large extent. The reader approaching the abstract for the 1st time cannot understand part of it. As an example, both in the abstract and elsewhere in the main text the definition of the "equivalent electric field strength" remains completely obscure and elusive.

**Ans:** Thank you for your comment. We apologize for our obscure expression, and we completely rewrite the abstract in the in the revised manuscript. Please see the following sentences in the revised manuscript: “In this work, the electro-coalescence process of three nanodroplets under a constant DC electric field is investigated via molecular dynamics simulations (MD), aiming to explore the electric manipulation of multiple droplets coalescence on the molecular level. The symmetrical and asymmetrical dynamic evolutions of electrocoalescence process can be observed. Our MD simulations show that there are two types of critical electric fields to induce the special dynamics. The chain configuration can be formed, when one of the critical electric field is exceeded, referred to as *E*_cc_. On the other hand, there is another critical electric field to change the coalescence pattern from complete coalescence to partial coalescence, the so-called *E*_cn_. Finally, we find that the use of the pulsed DC electric field can overcome the drawbacks of the constant DC electric field in the crude oil industry, and the mechanisms behind the suppressed effect of the water chain or non-coalescence are further revealed.”

(Please see Page 1, Lines 8-18.)

2. Introduction: This section is very poorly written and the English level is very scarce, in my opinion. I suggest extensive re-writing of this section. Moreover, the bibliographic apparatus presented in this Section is definitely incomplete. In the last decade, indeed, several important computational article dealing with water under electric fields have been reported in the literature [JCP 158, 184501 (2023); JCP 147, 031102 (2017); JCP 150, 074505 (2019)], just to cite a few. None of these important works is cited in the manuscript.

**Ans:** Thank you for your comment. We apologize for our poor expression, and we completely rewrite the section of the introduction in the revised manuscript. Additionally, we cite these literature in Refs. 20 and 26.

3. Methods: In this section the Authors state that "the dynamic electrocoalescence of three nanodroplet systems under a constant electric field is first investigated via MD simulations." However, they present the results of only one single system composed of three nanodroplets. The initial sentence is completely misleading.

**Ans:** Thank you for your comment. We apologize for our inaccurate statement, and we correct the statement in the revised manuscript. Please see the following sentences in the revised manuscript: “For the present work, we perform MD simulations to investigate dynamic evolution of coalescence process of three nanodroplets induced by external electric fields.”

(Please see Page 5, Lines 2-3.)

4. Methods: The Authors use the non-polarisable SPC/E water model in presence of strongly polarizing electric fields. Such an intrinsic and crucial limitation must be stressed both in the Methods section and throughout the manuscript as well. Of course, neglecting these important effects might limit the predictability level of the presented findings.

**Ans:** Thank you for your comment. We have pointed out that SPC/E model is non-polarisable in Simulation model and method in the revised manuscript: “It should be noted that the SPC/E water model is non-polarisable; therefore, the change of orientation of water molecules is the main effect of electric field.”

(Please see Page 6, Lines 6-7.)

5. Methods: The Authors state that they use "charged nanodroplets". However, they never specify how these are charged since they are composed of only neutral water and KCl (globally neutral) species. Please specify how these droplets result to be charged and how much charged they are! This is essential to figure out the macroscopic electrostatic force exerted by the field on them.

**Ans:** Thank you for your comment. As reviewer comment, the water together with KCl are globally neutral, whereas each atom contains a certain amount of charge. For the SPC/E water model, the charge of H atom is +0.4238, while it of O atom is -0.8476; for KCl, the charge of K atom is +1, while it of Cl atom is -1. This information has been added in the revised manuscript: “Here, the charge of K atom is +1, while it of Cl atom is -1” and “In the SPC/E model, the charge of H atom is +0.4238, while it of O atom is -0.8476.”

(Please see Page 5, Lines 15-16; Page 6, Lines 8.)

6. In Figure 5 all field strengths are in the order of 1-5 V/nm whereas in the text the Authors report one order of magnitude fields (0.1-0.5 V/nm). Please PAY ATTENTION on the strength of the field! One order of magnitude of difference may lead (and in general leads!) to completely different responses of molecular systems.

**Ans:** Thank you for your comment. We appologise for our typos. Figure 5 has been changed to Figure 6, and correct order of magnitude fields are marked in the revised manuscript.

7. Related to the previous point is the fact that a large fraction of the water molecules should be re-oriented by the field [JCPL 11, 8983 (2020); PCCP 21, 21205 (2019)]. Could the Authors comment more on this aspect and/or determine the amount of re-oriented water molecules? This is very important to understand how the field is capable of, e.g., increasing the total dipole moment inside the nanodroplets.

**Ans:** Thank you for your comment. Previous demonstrated that directional arrangement of water molecules occurs by presence of the applied electric field [R1, R2]. Beside the atoms being in the vicinity of added ions, almost all of oxygen atoms align along the opposite direction of the electric field for the water droplet except; however, the directional arrangement can be destroyed, and oxygen atoms are closer to sodium ions and hydrogen atoms are closer to chloride ions, as shown in Figure R1. The following sentences have been added in the revised manuscript: “Water molecules are subsequently polarized in the electric field circumstance, leading to the realignment of polarized molecules. Almost all atoms within water droplets rearrange in such a way, electropositive hydrogen atoms are oriented toward the electric field direction whereas electronegative oxygen atoms are against the electric field direction.”

(Please see Page 7, Lines 11-15.)

[R1] B. B. Wang, X. D. Wang, Y. Y. Duan, M. Chen, Molecular dynamics simulation on evaporation of water and aqueous droplets in the presence of electric field, International Journal of Heat and Mass Transfer, 73 (2014) 533-541.

[R2] Y. Okuno, M. Minagawa, H. Matsumoto, A. Tanioka, Simulation study on the influence of an electric field on water evaporation, Journal of Molecular Structure: THEOCHEM, 904 (2009) 83–90.

Figure R1. Snapshots of local particle distributions at *E*=0.8 V nm^-1^: (a) water droplet; (b) aqueous droplet with dissolved 120 NaCl molecules.

8. General comment: the results the Author report are interesting but rather qualitative and descriptive. Maybe, they could exploit the revision process to go deeper with some more quantitative analysis that would improve the quality of the findings reported in this work.

**Ans:** Thank you for your comment. We have carefully addressed your comments and with the help of your comments, our manuscript has got greatly improved. The following sentences have been added in the revised manuscript: “Interestingly, we find that the secondary droplet at the left side contains more number of water molecules compared with that for the right side. To explain this, we calculate hydration number at the time point when the secondary droplets generate to estimate stability of hydration effect, which is defined as the average number of water molecules in the first solvation shell, expressed as follow:

|  | $N_{\text{ion-O}}^{\text{sol}}=\rho_{\text{ion}}4\text{π}\int_{0}^{r_{\text{sol}}} g_{\text{ion-O}}(r)r^{2}\text{d}r$ | (2) |
| --- | --- | --- |

where *ρ*_ion_ is the number density of ions, *g*_ion-O_(*r*) is the ion radial distribution function, *r*_sol_ is the radius of the first solvation shell corresponding to the first valley in curve of *g*_ion-O_(*r*). As shown in **Fig. 8**, the first valley of *g*(*r*) appears at 3.45 Å for K^+^–O and at 3.95 Å for Cl^—^O. It indicates that, when electrocoalescence process produces some tiny daughter droplets, the corresponding hydration number for K^+^ and Cl^-^ are 6.32 and 7.20. Therefore, water molecules show a relative weak constraint force for K^+^ compared with that for Cl^-^, which determines molecular cluster with dissolved K^+^ is readily ejected from the primary droplet to form a larger secondary droplet.”

(Please see Page 12, Lines 17-21; Page 13, Lines 1-7.)

***We look forward to hearing from you regarding our revised manuscript, and we would be glad to respond to any further questions and comments that you may have. Thanks again for your valuable comments and suggestions.***
