# Supplementary material for: Coalescence of multiple droplets induced by a constant DC electric field
Source: PLoS One. 2024 Apr 9;19(4):e0300925. doi: 10.1371/journal.pone.0300925 (PMC11003697; doi:10.1371/journal.pone.0300925)
Supplement: Attachment — Submitted filename: Response to Reviewer #2.doc [file pone.0300925.s003.doc]

**Response to Reviewer #2**

The Authors have put some effort in addressing most, but not all, of the points I've raised during the previous round of review.

**Ans:** Thank you very much for your comments. We responded to your specific comments point by point below.

1. First of all, the English level of the manuscript remained relatively poor, even though some effort has been done in improving its readability. On the scientific perspective, I believe that the Authors should consider more seriously to include some relevant bilbiography I suggested in the previous round where the effects carried by electric fields in water are treated at the quantum level (see, e.g., [JCPL 11, 8983 (2020)]). Unfortunately, the Authors didn't take advantage of the revision process to include this aspect, which is tightly related to some of the results the Authors report here. In fact, from that work turns out the timescales and the dynamics associated with the re-orientation of the water dipoles under the field effect while considering the water molecules at the electronic and nuclear quantum level. Unfortunately, the Authors completely skip my suggestion.

**Ans:** Thank you for your comment. We apologize for our obscure expression, and we completely rewrite the manuscript in the in the revised manuscript. Please see the following sentences in the revised manuscript: “It is worth pointing out that the proton conductivity in liquid water at the nanoscale is significantly enhanced under the action of an external electric field [39]. Cassone provided a deep insights into the phenomenon of the enhanced proton conductivity and was ascribed to nuclear quantum effects [39]. For process of the filed-induced coalescence, the electrostatic force between two adjacent nanodroplets may be increased, which may directly lead to diverse results compared with that of electrocoalescence for macro-droplets: (Ⅰ) acceerative droplets coalescence, and reduction of critical electric field for (Ⅱ) chain configuration, *E*_cc_, and (Ⅲ) non-coalescence process.”

(Please see Page 13, Lines 13-20)

***We look forward to hearing from you regarding our revised manuscript, and we would be glad to respond to any further questions and comments that you may have. Thanks again for your valuable comments and suggestions.***
